# PCSK9 inhibitors reduces arterial stiffness in patients with acute coronary syndrome

**DOI:** 10.5937/jomb0-54773

**Published:** 2025-06-13

**Authors:** Liang Wang, Ruijie Wang, Tiantian Jiao, Linghao Xu, Endong Ji, Yuanzhen Jiang, Yuanqi Wang, Yehong Liu, Jiming Li

**Affiliations:** 1 Tongji University, School of Medicine, Shanghai East Hospital, Shanghai, China

**Keywords:** PCSK9 inhibitors, acute coronary syndrome (ACS), arterial stiffness, PCSK9 inhibitori, akutni koronarni sindrom (ACS), ukočenost arterija

## Abstract

**Background:**

This study focuses on uncovering the effects of proprotein convertase subtilisin/kexin type 9 (PCSK9) inhibitors in attenuating arterial stiffness in patients with acute coronary syndrome (ACS) and atherosclerosis.

**Methods:**

A total of 71 ACS patients were enrolled in this study from April 1, 2022, to June 31, 2022. Patients were randomly assigned to two groups: one group received statin therapy combined with PCSK9 inhibitors (Evolocumab 140 mg or Alirocumab 75 mg every two weeks) (n = 36), and the other group received statins alone (n = 35). All patients underwent measurements of lipid metabolism and arterial stiffness at baseline, 1 month, and 6 months after treatment initiation. Statistical power analysis indicated that the sample size of 71 patients provided sufficient power to detect significant differences.

**Results:**

After 1 month, the group treated with statins and PCSK9 inhibitors showed significantly greater reductions in total cholesterol (TC), triglycerides (TG), low-density lipoprotein (LDL), and lipoprotein(a) [Lp(a)] levels compared to the statin-only group (p = 0.027 and p = 0.021, respectively). By the 6-month follow-up, significant reductions were observed in pulse wave velocity (PWV) and ankle-brachial index (ABI) in the combination treatment group (p < 0.05). However, no significant differences were observed between Evolocumab and Alirocumab in terms of arterial stiffness improvement (p > 0.05). Statistical power was sufficient to detect these changes.

**Conclusions:**

The findings suggest that PCSK9 inhibitors, when combined with statins, not only improve lipid metabolism but also reduce arterial stiffness, offering potential benefits for vascular health in patients with ACS and atherosclerosis. Further studies with larger sample sizes and longer follow-up periods are necessary to confirm these results.

## Abstract (sr)

**Uvod:**

Ova studija se fokusira na otkrivanje efekata inhibitora proprotein konvertaze subtilizin/keksin tip 9 (PCSK9) na smanjenje ukočenosti arterija kod pacijenata sa akutnim koronarnim sindromom (ACS) i atero sklerozom.

**Metode:**

Ukupno 71 pacijent sa ACS je uključen u ovu studiju od 1. aprila 2022. do 31. juna 2022. Pacijenti su nasumično raspore|eni u dve grupe: jedna grupa je primala terapiju statinima u kombinaciji sa inhibitorima PCSK9 (Evolokumab 140 mg ili Alirokumab 75 mg svaka dva nedelja) (n = 36), a druga grupa je primala samo statine (n = 35). Svi pacijenti su podvrgnuti merenju metabolizma lipida i krutosti arterija na početku, 1 mesec i 6 meseci nakon početka lečenja. Statistička analiza snage pokazala je da je veličina uzorka od 71 pacijenta pružila dovoljnu snagu za otkrivanje značajnih razlika.

**Rezultati:**

Nakon mesec dana, grupa lečena statinima i PCSK9 inhibitorima pokazala je značajno veće smanjenje nivoa ukupnog holesterola (TC), triglicerida (TG), lipoproteina niske gustine (LDL) i lipoproteina(a) [Lp(a)] u poređenju sa grupa koja je koristila samo statine (p = 0,027 i p = 0,021, respektivno). Do 6-mesečnog praćenja, primećeno je značajno smanjenje brzine pulsnog talasa (PVV) i skočno-brahijalnog indeksa (ABI) u grupi koja je primala kombinovani tretman (p < 0,05). Međutim, nisu primećene značajne razlike između Evolokumaba i Alirokumaba u pogledu poboljšanja krutosti arterija (p > 0,05). Statistička snaga je bila dovoljna da otkrije ove promene.

**Zaključak:**

Nalazi sugerišu da inhibitori PCSK9, kada se kombinuju sa statinima, ne samo da poboljšavaju metabolizam lipida već i smanjuju krutost arterija, nudeći poten cijalne koristi za vaskularno zdravlje kod pacijenata sa ACS i atero sklerozom. Dalje studije sa većim uzorcima i dužim periodima praćenja su neophodne da bi se potvrdili ovi rezultati.

## Introduction

Acute coronary syndrome (ACS) is one of the most common chronic diseases that endanger human health. Its common risk factors include aging, smoking, dyslipidemia, diabetes, and hypertension. Despite treatment with drugs such as anti-thrombolysis, regulation of blood lipids, blood pressure and blood sugar, or revascularization therapy such as stent implantation and surgical bypass, patients with ACS are still at risk for recurrence of cardiovascular events.

More than 20% of patients have a risk of recurrence greater than 30% within 10 years, including myocardial infarction, stroke, or vascular death [1] [2], which is related to substandard regulation and control of blood lipids [3]. In recent years, there has been a focus on the concept of atherosclerosis as a chronic inflammatory disease [4]. Specifically, inflammation is believed to be a pathological manifestation of hypercholesterolemia and immune system dysfunction [5]. This concept may partially explain why atherosclerotic cardiovascular disease (ASCVD) remains a leading cause of death and disability-adjusted life-years despite lifestyle changes and the use of conventional lipid-lowering therapy (LLT) to reduce plasma cholesterol levels. Arterial stiffness (AS) is an independent predictor in cardiovascular disease (CVD) and adverse cardiovascular events [6]. AS results from dysregulation of elastin fibers and collagen, oxidative stress, mineral metabolism disorders, and low-grade inflammation [7], all of which lead to increased myocardial preload and decreased coronary perfusion pressure.

The measurement of pulse wave velocity (PWV) is a noninvasive and accurate method to evaluate AS in clinical practice. Over the past decade, more evidence has emerged showing the role of PWV as a potential marker for mechanical vascular injury, and its association with carotid atherosclerosis [8]. Moreover, PWV is a strong predictor of cardiovascular events and has been shown to correlate with increased intima-media thickness (IMT), suggesting it as a robust cardiovascular risk marker [9] [10].

The PCSK9 inhibitor (PCSK9-i) exerts an antiatherosclerosis effect by reducing the level of plasma low-density lipoprotein cholesterol (LDL-C) in the blood [11]. Mandraffino et al. demonstrated that the addition of PCSK9-i significantly improved PWV profiles in patients with FH [12].

In the lipid-lowering pharmacological strategy, the addition of PCSK9 inhibitors can effectively reduce LDL-C levels. PCSK9 inhibitors prevent the degradation of low-density lipoprotein receptors (LDL-R), thereby increasing their circulation on the surface of hepatocytes and reducing circulating LDL-C [13]. Another study demonstrated an independent association between PWV and the risk of carotid plaque formation [14]. A recent study has shown that PCSK9, regardless of LDL-C levels, can directly promote atherosclerosis progression by stimulating proinflammatory cytokine production and promoting oxidative stress in atherosclerotic lesions [9].

Despite these promising findings, there is limited data examining the relationship between PCSK9 inhibitors and changes in arterial stiffness, particularly in the context of ACS. Some studies have indicated that PCSK9 inhibitors not only lower lipid levels but also potentially influence vascular remodeling, including improvements in arterial stiffness. Therefore, the aim of this study was to assess changes in blood lipid levels and arterial stiffness in ACS patients treated with PCSK9 inhibitors, including their effect on PWV, in order to provide more clinical evidence for treating ACS patients with these medications.

## Materials and methods

### Study design and population

According to the emergency rapid diagnosis and treatment guidelines for ACS (7), ACS is defined as including ST-segment elevation myocardial infarction (STEMI), non-ST-segment elevation myocardial infarction (NSTEMI), and unstable angina pectoris (UAP). We considered a total of 71 ACS patients admitted to our hospital from April 1, 2022, to June 31, 2022, who underwent coronary angiography.

Patients were randomly assigned to either the treatment or control group using a simple randomization process. Randomization was not stratified by characteristics such as age or baseline lipid levels. All patients were included in the study based on several inclusion and exclusion criteria. Inclusion criteria consisted of being above 18 years old, meeting the diagnostic algorithm for STEMI, NSTEMI, UAP according to the guidelines, and having an LDL-C level > 1.4 mmol/L [7]. Exclusion criteria included clinical instability (hemodynamic or electrical), severe renal insufficiency (EGFR < 30 mL/min), previous use of PCSK9-i, treatment with systemic steroids or systemic cyclosporine within the past 3 months, active infection or major hematologic, metabolic, or endocrine dysfunction, active malignancy requiring treatment, pregnancy status, persistent atrial fibrillation, severe aortic insufficiency or stenosis; peripheral artery disease (ankle-brachial index 0.9 or history of lower extremity bypass and/or endovascular treatment).

While the exclusion of patients with severe renal insufficiency and severe aortic insufficiency may limit the generalizability of our findings, these conditions were excluded to ensure patient safety and minimize confounding factors, as they are commonly associated with higher risks of adverse outcomes in ACS.

All patients were voluntarily divided into treatment groups. A combination of PCSK9 inhibitors (Evolocumab 140 mg or alirocumab 75 mg) and statins (atorvastatin 20 mg or rosuvastatin 10 mg) was administered subcutaneously every two weeks to one group (n = 36). The control group received monotherapy with statins only (atorvastatin 20 mg or rosuvastatin 10 mg) (n =35). All patients received the same standard of care, including aspirin, clopidogrel, angiotensin-converting enzyme inhibitors/angiotensin receptor blockers, beta-blockers, and statins unless contraindicated.

The study was conducted in accordance with the principles of the Declaration of Helsinki, and the ethics committee of Shanghai East Hospital approved the study design and permitted the use of clinical data; all patients who met the inclusion criteria signed an informed consent.

### Data collection

The general data of patients were collected, including gender, age, history of hypertension and diabetes, smoking history, and statin treatment (including atorvastatin and rosuvastatin) before or after PCSK9-i. During the present study, blood was taken to examine fasting blood glucose, glycated hemoglobin A1c (HbA1c), N-terminal pro-brain natriuretic peptide (NT-proBNP), troponin levels, total cholesterol (TC), triglyceride (TG), low-density lipoprotein (LDL), high-density lipoprotein (HDL), sd- LDL levels, lipoprotein(a) levels. Additionally, PWV and ankle brachial index (ABI) were measured at the beginning of treatment as well as 1 month and 6 months after treatment.

Blood samples were collected from all patients after an 8-hour fasting period and sent to our hospital’s laboratory for analysis. Blood glucose and blood lipid levels were measured using the Roche biochemistry analyzer 702, while HbA1c was measured using the MQ6000 hemoglobin instrument. PWV and ABI measurements were taken using the Omron arteriosclerosis detector (BP-203RPEIII) while patients were in a resting state, with all measurements performed by the same professional physician to minimize bias. All procedures strictly followed the instructions.

### Statistical analysis

The data were analyzed using the Statistic Package for Social Science (SPSS) 25.0 statistical package (IBM, Armonk, NY, USA). Count data was described as frequency (%). Continuous variables were presented as mean ± standard deviation (SD) if normally distributed, and as medians and interquartile ranges (IQR) if non-normally distributed. Comparisons of differences between two groups were performed using an unpaired T-test or a Mann–Whitney U test for continuous variables, and a chi-squared test or Fisher’s exact test for dichotomous variables, when appropriate. The Kolmogorov–Smirnov test was used to determine whether the distribution was normal or non-normal. A power analysis was conducted prior to the study, and the sample size was determined based on the expected effect size and desired power level. A two-sided *p*<0.05 was considered statistically significant.

## Results

The clinical characteristics of the subjects enrolled in the study are provided in Table 1. There were no significant differences in gender, height, weight, blood glucose, and blood lipid levels between the two groups before treatment. However, significant differences were observed in age (p = 0.028), with the PCSK9 inhibitor group being younger on average than the control group.

**Table 1:** The difference of clinical characteristics between the two groups.

	PCSK9-i+ Statins (n=36) <br>Mean ± SD	Statins (n=35) <br>Mean ± SD	p-Value
Age (year)	63.86±12.15	69.80±9.27	0.028*
female	8	11	0.430
male	28	24	
BMI (m^2^/kg)	25.06±2.75	24.99±2.65	0.913
LVEF (%)	49.69±6.53	50.83±6.960	0.297
HbA1c (%)	6.41±1.29	6.45±0.93	0.499
Tnt (ng/mL)	3.62±4.02	2.57±3.10)	0.284
NTproBNP (ng/L)	2250.90±4838.72	1449.27±2060.96	0.868
TC (mmol/L)	4.63±1.08	4.31±1.20	0.243
TG (mmol/L)	1.63±0.86	1.69±0.73	0.421
HDL (mmol/L)	1.05±0.26	1.06±0.29	0.904
LDL (mmol/L)	3.04±0.90	2.93±0.83	0.476
sd LDL (mmol/L)	0.90±0.46	0.71±0.41	0.062
Lp (a) (nmol/L)	62.72±69.62	44.35±51.86	0.233
LPWV	1438.11±171.73	1427.51±164.88)	0.516
RPWV	1492.69±206.64	1455.97±166.03	0.314
LABI	1.10±0.11	1.10±0.12	0.787
RABI	1.11±0.09	1.09±0.12	0.580

After a 1-month follow-up, the levels of TC, TG, HDL, and LDL-C in the control group (patients who only used statins) decreased. By 6 months, there were significant decreases in LVEF and NTproBNP levels; however, there were no major differences observed in Lp(a), PWV, and ABI (p-values for these parameters are shown in Table 2).

**Table 2:** Clinical characteristics for ACS study group (n=71).

Age (years)	66.8±11.2
Male/Female (n)	52/19
BMI (kg/m^2^)	25.0±+2.7
Coronary heart disease, n (%)	12 (16.9%)
Diabetes mellitus, n (%)	18 (25.4%)
Heart failure, n (%)	4 (5.6%)
Stroke history, n (%)	2 (2.8%)
Hypertension, n (%)	40 (56.3%)
Smoking history, n (%)	35 (49.3%)
LVEF, %	50.25±6.72
HbA1c, %	6.43±1.12
TnT, ng/mL	3.10±3.61
NTproBNP, ng/L, <br>median [95% CI]	1855.73 <br>[972.23–2739.23]
STEMI, n (%)	46 (64.8%)
NSTEMI, n (%)	15 (21.1%)
Unstable angina, n (%)	10 (14.1%)

The levels of LVEF, NT-proBNP, TC, TG, LDL-C, and Lp(a) in the test group with PCSK9 inhibitors and statins had also decreased after 1 month. Compared with the control group, TC and LDL-C showed significant changes (p = 0.027 and p = 0.021, respectively). In the 6-month follow-up period, these changes became even more remarkable. Additionally, PWV and ABI were found to have decreased as well. The observed decrease in PWV and ABI indicates an improvement in arterial stiffness, but it would be valuable to consider the clinical relevance of these changes. Specifically, a clinically meaningful change in PWV is generally considered to be a reduction of at least 1–2 m/s, while a significant improvement in ABI would be a change of 0.1 or more, which corresponds to a reduction in cardiovascular risk. These changes, although statistically significant, may suggest clinical benefits in terms of reducing vascular injury and improving long-term cardiovascular outcomes.

Although there was no significant change in PWV after just one month of treatment with PCSK9 inhibitors and statins (Table 3, p = 0.516 for LPWV and p = 0.314 for RPWV), a comparison between alirocumab and evolocumab revealed that only the RPWV decrease in patients treated with alirocumab was statistically significant after a 6-month follow-up (p = 0.031) (Table 4). This result may reflect the potential variability in patient response to different PCSK9 inhibitors, although both evolocumab and alirocumab are part of the same drug class. Given that both drugs target the same PCSK9 pathway, it is possible that the observed differences are due to individual patient variability or pharmacokinetic differences between the two agents. It would be important for future studies to explore whether this difference has clinical implications for treatment choice.

**Table 3:** The indicators of lipid metabolism and AS between the two groups.

Indicators	PCSK9-i+ Statins					Statins				
	Mean ± SD					Mean ± SD				
	Baseline <br>(n=36)	1 month <br>(n=32)	6 months <br>(n=28)	p -Value <br>(1month <br>vs. <br>Baseline)	p -Value <br>(6months <br>vs. <br>1month)	Baseline <br>(n=35)	1 month <br>(n=34)	6 months <br>(n=33)	p-Value <br>(6 months <br>vs. <br>Baseline)	p -Value <br>(6 months <br>PCSK9 i <br>+ Statins <br>vs. Statins)
LVEF (%)	49.69 ±6.53	50.73± 6.90	52.61± 6.20	0.016	0.002	50.83± 6.96	51.91± 5.45	53.29± 4.61	0.004	0.862
NTproBNP <br>(ng/L)	2250.90 ±4838.72	993.92± 1937.25	607.30± 1685.09	0.012	0.000	1449.27± 2060.96	1068.86 ±1710.44	695.89± 832.13	0.004	0.001
TC <br>(mmol/L)	4.63± 1.08	3.39± 0.71	2.61± 0.60	0.000	0.000	4.31± 1.20	3.79± 0.81	3.71± 0.69	0.000	0.000
TG <br>(mmol/L)	1.63± 0.86	1.45± 0.54	1.31± 0.40	0.044	0.005	1.69± 0.73	1.51± 0.65	1.44± 0.51	0.021	0.312
HDL <br>(mmol/L)	1.05± 0.26	1.08± 0.29	1.22± 0.25	0.126	0.000	1.06± 0.29	1.16± 0.27	1.20± 0.25	0.000	0.347
LDL-C <br>(mmol/L)	3.04± 0.90	1.83± 0.67	1.26± 0.46	0.000	0.000	2.93± 0.83	2.22± 0.57	2.04± 0.57	0.000	0.000
Lp(a) <br>(nmol/L)	62.72± 69.62	37.85± 33.28	32.64± 24.13	0.009	0.060	44.35± 51.86	42.63 ±45.55	37.23± 26.68	0.986	0.383
sdLDL <br>(mmol/L)	0.90± 0.46	0.78± 0.34	0.66± 0.34	0.002	0.001	0.71± 0.41	0.76± 0.32	0.78± 0.30	0.133	0.064
LPWV	1438.11 ±171.74	1432.39± 171.59	1295.37± 121.35	0.100	0.000	1427.51± 164.88	1431.41 ±157.16	1429.59 ±151.45	0.743	0.000
RPWV	1492.69 ±206.65	1483.03± 200.97	1345.66 ±178.39	0.057	0.000	1455.97 ±166.03	1448.41 ±154.00	1447.82± 149.03	0.221	0.021
LABI	1.10± 0.11	1.09± 0.10	1.05± 0.06	0.002	0.000	1.10± 0.12	1.08±0.11	1.08± 0.10	0.984	0.050
RABI	1.11± 0.09	1.12± 0.14	1.08± 0.12	0.002	0.000	1.09± 0.12	1.09±0.11	1.09± 0.09	0.585	0.089

**Table 4:** The indicators of lipid metabolism and AS between with Evolocumab or Alirocumab.

Indicators	Evolocumab (n=22) Mean ± SD	Alirocumab (n=6) Mean ± SD	p-Value (Repatha vs. Praluent)
LVEF (%)	53.32±3.97	50.00±11.45	0.849
NTproBNP (ng/L)	217.85±185.19	2035.31±3474.91	0.643
TC (mmol/L)	2.63±0 .63	2.55±0.55	0.806
TG (mmol/L)	1.32±0.43	1.28±0.32	0.806
HDL (mmol/L)	1.20±0.28	1.29±0.13	0.259
LDL-C (mmol/L)	1.32±0.45	1.02±0.50	0.194
Lp(a) (nmol/L)	33.91±25.61	28.00±18.90	0.764
sdLDL (mmol/L)	0.71±0.34	0.48±0.28	0.157
LPWV	1314.07±105.80	1220.57±157.86	0.246
RPWV	1377.75±166.87	1217.29±176.06	0.028
LABI	1.05±0.07	1.04±0.06	0.505
RABI	1.08±0.13	1.06±0.04	0.984

We believe that there is a lack of significant difference between the different types of PCSK9 inhibitors used in patients; however, due to insufficient patient numbers and short follow-up time, this study cannot provide an accurate conclusion regarding the differences between these two types of PCSK9 inhibitors. Nevertheless, the observation of changes in PWV and ABI provides some insight into their potential for improving vascular health in ACS patients. There was no significant difference observed in the improvement of PWV and ABI among STEMI, NSTEMI, and UAP patients in the PCSK9 inhibitor group (p-values for these comparisons are available in Table 5). Similarly, there was no significant difference observed in the improvement of PWV and ABI before and after treatment among STEMI, NSTEMI, and UAP patients in the statins group (Table 6, p = 0.178 for PWV and p = 0.243 for ABI).

**Table 5:** The indicators of PWV and ABI between the two groups.

		Statins <br>Mean ± SD			p-Value	PCSK9-i + Statins <br>Mean ± SD			p-Value
		STEMI <br>n=19	NSTEMI <br>n=8	UA <br>n=8		STEMI <br>n=27	NSTEMI <br>n=7	UA <br>n=2	
LPWV	Baseline	1408.21± 129.68	1517.63± 262.59	1383.25± 85.53	0.649	1453.15± 168.94	1391.57± 199.29	1398.00± 165.46	0.744
	1 month	1406.58± 118.91	1524.38± 246.46	1392.57± 85.58	0.653	1449.07± 169.47	1383.14± 193.92	1379.50± 167.58	0.856
	6 months	1401.84± 109.15	1525.63± 240.04	1395.14± 84.04	0.651	1317.35± 110.29	1216.57± 145.72	1285.50± 113.84	0.247
RPWV	Baseline	1434.79± 133.70	1483.38± 226.03	1478.88± 184.85	0.871	1508.59± 201.10	1379.57± 189.16	1674.00± 257.39	0.256
	1 month	1437.74± 130.01	1496.88± 228.63	1422.00± 120.18	0.797	1501.07± 197.02	1379.86± 191.86	1600.50± 269.41	0.385
	6 months	1433.11± 121.29	1502.75± 222.42	1425.00± 122.45	0.775	1369.65± 168.56	1225.57± 161.37	1454.00± 275.77	0.229
LABI	Baseline	1.09± 0.11	1.15± 0.13	1.04± 0.13	0.391	1.11± 0.11	1.11± 0.06	0.95± 0.23	0.558
	1 month	1.07± 0.09	1.12± .15	1.05± 0.14	0.516	1.10± 0.09	1.09± 0.05	0.95± 0.20	0.586
	6 months	1.08± 0.08	1.11± 0.11	1.06± 0.12	0.758	1.06± 0.06	1.03± 0.05	0.97± 0.06	0.086
RABI	Baseline	1.07± 0.11	1.10± 0.16	1.13± 0.08	0.474	1.12± 0.10	1.08± 0.04	1.13± 0.11	0.494
	1 month	1.07± 0.11	1.09± 0.13	1.14± 0.09	0.442	1.14± 0.16	1.08± 0.05	1.10± 0.12	0.467
	6 months	1.07± 0.08	1.10± 0.12	1.13± 0.07	0.279	1.09± 0.13	1.04± 0.04	1.03± 0.05	0.312

**Table 6:** The analysis of therapeutic changes in blood lipids, PWV, and ABI.

		Statins Mean ± SD	PCSK9-i + Statins Mean ± SD	p-Value
ΔTC (mmol/L)	1month	0.51±1.24	1.43±1.57	0.0001
	6months	0.60±0.94	2.60±1.59	0.0001
ΔTG (mmol/L)	1month	0.19±0.47	0.26±0.67	0.890
	6months	0.26±0.59	0.62±0.80	0.104
ΔLDL (mmol/L)	1month	0.71±0.68	1.31±1.21	0.009
	6months	0.88±0.84	2.06±1.09	0.0001
ΔLPWV	1month	37.00±236.18	5.72±27.42	0.357
	6months	38.77±237.11	178.72±196.54	0.0001
ΔRPWV	1month	48.94±313.07	9.67±24.97	0.045
	6months	49.51±313.64	184.42±187.17	0.0001
ΔLABI	1month	0.05±0.22	0.02±0.03	0.110
	6months	0.05±0.21	0.08±0.17	0.001
ΔRABI	1month	0.03±0.20	-0.01±0.18	0.203
	6months	0.04±0.20	0.06±0.23	0.002

## Discussion

PCSK9 was initially considered a type of convertase, belonging to the secretory subtilisin enzyme family as its ninth member. It is primarily expressed in kidney, liver, and intestinal tissues and is known to regulate apoptosis in human nervous system cells [15].

Recent studies have found that PCSK9 inhibitors can induce hyperlipidemia and lead to atherosclerosis by promoting lysosomal degradation of LDL-R in the liver, which hinders the process of LDL-C clearance. PCSK9 inhibitors can bind to PCSK9, preventing it from binding to LDL-R and avoiding the decomposition of LDL-R, thereby increasing the uptake of LDL-C in the liver and reducing its concentration in the blood [11]. Both alirocumab and evolocumab, which are PCSK9 inhibitors, have been approved by the Food and Drug Administration (FDA) for treating patients with familial hypercholesterolemia, statin intolerance or contraindication, and ASCVD. The effect of PCSK9 inhibitors on lowering LDL-C has been unanimously recognized by the academic community; however, more research data is needed to understand their effects on other lipid components and arterial stiffness.

In the management of ACS patients, achieving optimal lipid control remains a significant challenge. Despite the use of statins, which are considered the cornerstone of LLT, a substantial proportion of ACS patients fail to reach the recommended LDL-C targets. This is due to several factors, including statin intolerance, inadequate response to statin therapy, and the complex lipid profiles seen in ACS patients. In this context, the addition of PCSK9 inhibitors to statin therapy has emerged as a promising strategy to further lower LDL-C levels and improve overall lipid management. PCSK9 inhibitors have shown the ability to reduce LDL-C beyond the effects of statins, addressing the unmet need for more effective lipidlowering options.

Fourier et al. [16] demonstrated that evolocumab can reduce LDL-C levels by approximately 59% compared to placebo and decrease the incidence rate of primary endpoint events in patients with multiple coronary artery disease by about 21%. In addition to its evident effect on lowering LDL-C, evolocumab also affects other lipoprotein metabolism. Odyssey EAST reported that alirocumab reduced all atherogenic lipoproteins, including a significant decrease of Lp(a) by 30.3% compared to baseline levels at 24 weeks [17]. Our study’s results are consistent with the FOURIER and Odyssey EAST studies, showing significant reductions in LDL-C, Lp(a), and sd-LDL levels after 1 and 6 months of treatment with the PCSK9 inhibitors alirocumab or evolocumab. However, no significant changes in Lp(a) and sd-LDL levels were observed in the control group. While lowering LDL-C remains crucial for patients with coronary heart disease, increasing attention is being given to Lp(a) and sd-LDL levels. Lp(a), which contains all atherogenic components of LDL, increases the risk of ASCVD events through various mechanisms [18]. Numerous studies have shown a significant correlation between elevated Lp(a) levels and myocardial infarction, stroke, and peripheral artery diseases. Furthermore, there is a positive correlation between CVD risk rates and Lp(a) levels [19] [20] [21]. In contrast, sd-LDL has a greater potency to be atherogenic than other LDL subcomponents and is therefore a better predictor of CVDs than LDL-C. Free sd-LDL in the circulation has a strong ability to penetrate the arterial intima, contributing to the formation of atherosclerotic plaques. It is also more prone to various atherogenic modifications such as deacetylation, glycosylation, and oxidation, which enhance its atherogenic properties and contribute to inflammatory processes associated with CVDs [22]. There are few effective methods to reduce Lp(a) and sd-LDL levels in clinical practice. Our study now shows that PCSK9 inhibitors significantly reduced these levels, suggesting that they may serve as an effective treatment for CVDs. After 1 month of PCSK9-i treatment, TC and LDL levels decreased significantly; however, there was no obvious improvement in blood lipid levels in patients treated with statins. We found that the treatment compliance rate among patients treated with PCSK9- i was 89.3%, while the follow-up rate among patients treated with statins was 67.5%. This difference may be due to the lower efficacy of statins, leading to poorer patient cooperation and follow-up (Table 6).

Unfavorable functional and structural changes in the vascular intima are the main reasons for increased AS, including extracellular matrix degeneration, collagen deposition and cross-linking, elastin depletion and fragmentation, proliferation of vascular smooth muscle cells, macrophage and monocyte infiltration, inflammation, and endothelial dysfunction [23]. These changes will reduce the capacity of the arterial system to cope with blood pressure changes and lead to an increase in aortic systolic pressure and pulse after increased arterial stiffness. This will subsequently increase the load on the myocardium, especially the left ventricular afterload, leading to left ventricular cell hypertrophy and an increase in oxygen demand within myocardial cells [24]. Additionally, aortic stiffness causes an increased velocity of the forward pressure wave which promotes early arrival of the reflected pressure wave during systole. This results in a decrease in coronary perfusion pressure and myocardial oxygen delivery during diastole, leading to myocardial ischemia [25]. Therefore, an imbalance between oxygen supply to the coronary arteries can make the myocardium more susceptible to ischemia.

The assessment of arterial stiffness (AS) can be ndone using various methods, but the most commonly used method is PWV. PWV has strong prognostic value in predicting cardiovascular events and all-cause mortality. It is considered the “gold standard” for assessing arterial stiffness due to its simplicity, noninvasiveness, reproducibility, and proven predictive value in epidemiological and clinical studies. Ankle-Brachial Index (ABI) is a simple yet valuable diagnostic technique that calculates the ratio of the highest systolic blood pressure in the ankle to that in the brachial artery. Moreover, ABI can be utilized for primary prevention by identifying and managing coronary artery disease (CAD) in symptomatic or asymptomatic patients at all levels of medical institutions [26]. A seven-year longterm follow-up study demonstrated an association between abnormal ABI and increased incidence of major cardiovascular events among patients with severe coronary heart disease [27].

Our study is consistent with the results of a study that demonstrated significant improvement in arterial stiffness in patients with familial hypercholesterolemia after 6 months of treatment with PCSK9 inhibitors [28]. Toscano et al. found that reducing plasma levels of PCSK9 was associated with mechanical vascular improvement following PCSK9 inhibitor therapy [29] [30]. However, our study revealed no significant differences between the effects of evolocumab and alirocumab on arterial stiffness. This lack of significant differences is noteworthy, especially considering that these two agents, while both PCSK9 inhibitors, differ in their mechanisms of action and pharmacokinetics. Evolocumab is a fully human monoclonal antibody with a longer half-life and less frequent dosing, whereas alirocumab, a fully humanized monoclonal antibody, is administered more frequently. These differences in pharmacokinetics might have contributed to the similar clinical outcomes observed in terms of arterial stiffness reduction in our study. This finding suggests that, despite their slight differences in pharmacokinetics and mechanisms, both PCSK9 inhibitors may provide similar clinical benefits for improving arterial stiffness in ACS patients.

We observed a statistically significant reduction in ABI after 1 month of PCSK9 inhibitor treatment, with further significant improvements at 6 months, along with a reduction in PWV. Moreover, both PWV and ABI were significantly improved in patients with STEMI, NSTEMI, or UAP following PCSK9 inhibitor treatment, with no significant difference in the effectiveness of the improvements between evolocumab and alirocumab. This suggests that PCSK9 inhibitors play a role in improving arterial stiffness and may provide a novel approach for the treatment of future ACS patients.

However, several limitations of our study should be acknowledged. The follow-up period of 6 months was relatively short, which may limit the ability to assess the long-term effects of PCSK9 inhibitors on arterial stiffness and overall cardiovascular outcomes. A longer follow-up period would provide valuable insights into the sustained benefits of these treatments. Additionally, this was a single-center study, which may limit the generalizability of the findings to a broader population. Multi-center, longer-term studies are necessary to validate these results and further explore the mechanisms by which PCSK9 inhibitors improve vascular function. Moreover, while the sample size was reasonable for detecting short-term effects, it may not have been large enough to capture long-term outcomes or subtle differences between groups. The potential impact of other factors, such as endothelial function or inflammatory pathways, which might contribute to improvements in arterial stiffness, should be explored in future studies.

Lastly, although the study suggests that PCSK9 inhibitors could be a novel treatment approach for improving arterial stiffness in ACS patients, long-term safety and efficacy data from larger, multi-center studies will be crucial to confirm these findings and establish the role of PCSK9 inhibitors in broader cardiovascular disease management.

## Conclusion

Taken together, our research suggests that PCSK9 inhibitors can not only significantly reduce LDL-C, Lp(a), and sd-LDL levels but also improve PWV and ABI in ACS patients, thereby improving AS. However, these findings should be interpreted with caution, as further, larger-scale trials are needed to confirm the effects of PCSK9 inhibitors on arterial stiffness and to investigate their long-term impact on major adverse cardiovascular events.

## Dodatak

### Funding

Top-level Clinical Discipline Project of Shanghai Pudong District Grant (Award Number: PWYgf 2021- 01).

### Availability of data and materials

All data were obtained from patients at Shanghai East Hospital. Thanks to Shanghai East Hospital, the biospecimens and data used for this study were provide by Shanghai East Hospital. The datasets generated and/or analysed during the current study are not publicly available due to involving the privacy of patients, but are available from the corresponding author on reasonable request.

### Ethics approval

The study was conducted according to the principles of the Declaration of Helsinki, and the ethics committee of Shanghai East Hospital approved the study design and allowed the use of clinical data.

### Consent to participate

An informed consent was signed by all patients who met the inclusion criteria.

### Consent for publication

Not applicable.

### Authors’ contributions

Liang Wang wrote the manuscript and researched data. Ruijie Wang and Tiantian Jiao researched data. Linghao Xu and Endong Ji researched data and contributed to discussion. Yuanzhen Jiang and Yuanqi Wang contributed to the discussion. Yehong Liu reviewed and edited the manuscript. Jiming Li is the guarantor of this work, had full access to all the data, and takes full responsibility for the integrity of data and the accuracy of data analysis.

### Acknowledgements

All data were obtained from patients at Shanghai East Hospital. Thanks to Shanghai East Hospital, the biospecimens and data used for this study were provide by Shanghai East Hospital.

### Conflict of interest statement

All the authors declare that they have no conflict of interest in this work.
